# Late post-operative recurrent osteosarcoma: Three case reports with a review of the literature

**DOI:** 10.3892/ol.2013.1322

**Published:** 2013-04-29

**Authors:** XIUCHUN YU, SUJIA WU, XUQUAN WANG, MING XU, SONGFENG XU, YE YUAN

**Affiliations:** 1 Department of Orthopedics, The General Hospital of Jinan Military Command, Jinan, Shandong 250031;; 2 Department of Orthopedics, The General Hospital of Nanjing Military Command, Nanjing, Jiangsu 210002;; 3 Department of Orthopedics, Xinan Hospital of The Third Military Medical University, Chongqing 400038, P.R. China

**Keywords:** osteosarcoma, late recurrence, treatment, prognosis

## Abstract

The aim of the present study was to investigate the clinical characteristics and treatment of late recurrent osteosarcoma following surgery. The cases of three patients with late recurrent osteosarcoma, who were treated at the General Hospital of Jinan Military Command, General Hospital of Nanjing Military Command and Xinan Hospital of The Third Military Medical University, were analyzed retrospectively. Furthermore, 10 cases of late recurrent osteosarcoma were retrieved from the literature. In total, eight male and five female cases were selected for the present study. The mean age at recurrence was 25.56 years (range, 13–42 years). The locations of the osteosarcomas were as follows: five cases in the distal femur, two cases in the distal tibia and acetabulum, respectively, and one case in the proximal tibia (the remaining cases were not described). The tumors were histologically classified into three cases of fibroblastic, two cases of traditional-type; two cases of mixed-type and one case each of osteoblastic-, chondroblastic- and telangiectasia-type osteosarcoma (the remaining cases were not described). The mean recurrence time following surgery was 10.02 years (range, 5.2–19.3 years). With regard to the treatment modalities, five patients accepted surgery and chemotherapy, one patient accepted surgery and radiotherapy, two patients accepted surgery alone and one patient did not complete the treatment (the remaining cases were not described). From the 12 cases that were followed-up for between 0.5 and 4.7 years (mean, 2.28 years), one case was lost to follow-up, six patients survived (up to 4.5 years) and six patients succumbed to their condition (0.6–4.7 years). The present study highlights the fact that more focus should be placed upon the long-term follow-up of patients with osteosarcoma. A follow-up is required once every six months, from five years after the diagnosis. The abnormal changes in the surgical site should also receive further attention, in addition to the pulmonary and systemic metastases. Following a diagnosis of late post-operative recurrence, surgery and post-operative chemotherapy are commonly used in clinical treatment, however, the clinical outcome of osteosarcoma requires further observation.

## Introduction

Osteosarcoma is the most common primary malignant bone tumor. Although the clinical outcome has significantly improved with the application of neoadjuvant chemotherapy, the recurrence of osteosarcoma remains a common post-operative complication, often resulting in treatment failure. It is generally recognized that the post-operative recurrence of osteosarcoma may occur within two years post-surgery, with the possibility of recurrence decreasing gradually with the prolongation of the follow-up time. However, although it is extremely rare, a recurrence may also occur five years subsequent to the treatment (1), which is defined as a late recurrence of osteosarcoma. The present study retrospectively analyzed three patients with late recurrent osteosarcomas who were treated at the General Hospital of Jinan Military Command, General Hospital of Nanjing Military Command and Xinan Hospital of The Third Military Medical University (Chongqing, China). Furthermore, 10 similar cases from the literature were comprehensively reviewed in order to determine the correct diagnosis and treatment for this disease. This study was approved by the ethics committee of the General Hospital of Jinan Military Command. Written informed consent was obtained from all patients.

## Case reports

### Case 1

**Figure 1.**
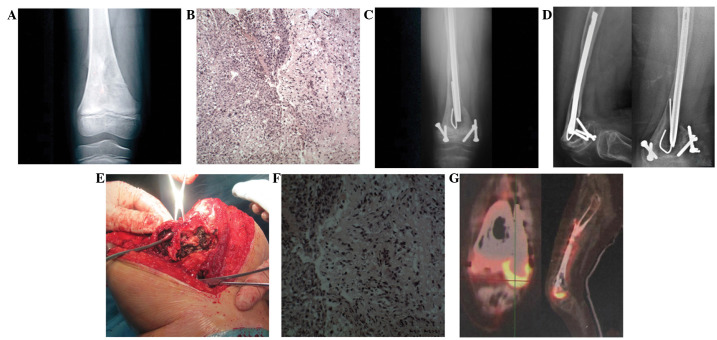
A female patient (case 1) with late recurrent osteosarcoma at 6.5 years post-surgery. (A) Radiography revealing a mixed osteolytic-osteoblastic lesion with periosteal reaction of the right distal femur and a shadow of a soft tissue mass. (B) Histological examination of the biopsy specimen demonstrating the diagnosis of osteosarcoma (HE; magnification, ×10). (C) X-ray showing good healing between the inactivated and host bones at 6 months following an en bloc resection of the tumor and an inactivated bone replantation with preservation of the epiphysis. (D) X-ray showing the healing between the inactivated bone and femoral shaft at 6.5 years post-surgery. The diameter of the affected femur was thinner than that of the contralateral one and a forward protrusion between the inactivated bone and the preserving epiphysis was present. (E) During the second surgery, granulation-like tissue was identified in the medial femoral condyle. (F) A post-operative pathological examination of the specimens indicated that the curetted tissue was that of an osteosarcoma (HE; magnification, ×20). (G) PET/CT examination showing abnormal bone metabolism at the right distal femoral condyle. HE, hematoxylin and eosin; PET/CT, positron emission tomography/computed tomography.

A six-year-old female was admitted to the General Hospital of Jinan Military Command on June 9, 2005 due to right knee pain that had lasted for one and a half months, with no significant pain at night. A physical examination revealed a 6×4-cm mass in the medial-posterior side of the right distal femur, with obvious tenderness to the area and a poorly-defined border. X-ray imaging (Fig. 1A) revealed a mixed osteolytic-osteoblastic lesion with a periosteal reaction of the right distal femur and a shadow of a soft tissue mass. MRI showed a mixed high and low signal intensity lesion in the right distal femur, with evidence of a soft tissue mass, but no tumor invasion of the epiphysis. A needle aspiration biopsy was performed and a histological examination of the specimen confirmed the diagnosis of osteosarcoma (Fig. 1B). The patient then received two courses of pre-operative DIA chemotherapy [doxorubicin (ADR), 30 mg/m^2^ × 3; ifosfamide (IFO), 2 g/m^2^ × 5; and cisplatin (DDP), 120 mg/m^2^]. The patient’s pain symptoms disappeared following chemotherapy. The X-ray examination showed osteolytic damage to the right distal femur with a clear boundary. A tumor bone calcification shadow was visible in the medullary cavity, but the surrounding soft tissue mass had disappeared. MRI showed a significantly reduced lesion in the right distal femur and an evident disappearance of the soft tissue swelling, without tumor invasion of the epiphysis. On September 1, 2005, under epidural anesthesia, the patient underwent an en bloc resection of the tumor and an inactivated bone replantation with preservation of the epiphysis. The incision healed and no complications occurred. A post-operative histological examination of the specimen confirmed the evident degeneration and necrosis of the osteosarcoma cells. Post-operative chemotherapy was begun at two weeks post-surgery, with the same regimen as the pre-operative chemotherapy, and lasted for six courses with three-week intervals. A post-operative X-ray following six months of treatment showed good healing between the inactivated and host bones (Fig. 1C), and the knee was able to achieve 90 degrees of flexion. However, at two years post-surgery, the affected limb began to shorten, which gradually limited the knees function. On February 22, 2012, 6.5 years after the initial surgery, the patient was readmitted into hospital due to one week of pain in the affected knee. The patient was unable to walk due to a goose-shaped deformity of the right knee. While there was no obvious tenderness to the area, the patient was unable to straighten the affected knee. The affected limb was 7 cm shorter than the contralateral one. An X-ray examination confirmed good healing between the inactivated bone and the femoral shaft (Fig. 1D). However, the diameter of the affected femur was thinner than that of the contralateral one and bone fragmentation was observed at the healing site, connecting the inactivated bone and the preserved epiphysis, which formed a forward protrusion. Emission computed tomography (ECT) and lung CT scans showed no abnormalities. The patient was then diagnosed with a nonunion following inactivated bone replantation with preservation of the epiphysis for osteosarcoma of the right distal femur. On March 4, 2012, an en bloc resection of the inactivated tumor bone and an allograft bone transplantation were performed under general anesthesia. Granulation-like tissue was observed intraoperatively in the medial femoral condyle. This was considered to be a reaction of the surrounding bones to the screw and was subsequently removed completely (Fig. 1E). However, a post-operative pathological examination of the specimen indicated that the curetted tissue was that of osteosarcoma (Fig. 1F), with the same histology as the specimen from the initial diagnosis. On March 15, 2012, a positron emission tomography (PET)/CT examination showed abnormal bone metabolism at the right distal femoral condyle (Fig. 1G), which was diagnosed as a recurrence of the osteosarcoma of the right distal femur. On March 23, 2012, under general anesthesia, the entire right knee joint was removed due to osteosarcoma at the femoral condyle and an inactivated allograft bone replantation and arthrodesis were performed. The incision stitches were removed at 14 days post-surgery and the wound healed well. Chemotherapy was initiated two weeks after the surgery, using the original regimen, and stopped following two courses of treatment due to the occurrence of cardiac hypertrophy. There was no recurrence or metastasis within a follow-up period of 8 months.

### Case 2

**Figure 2.**
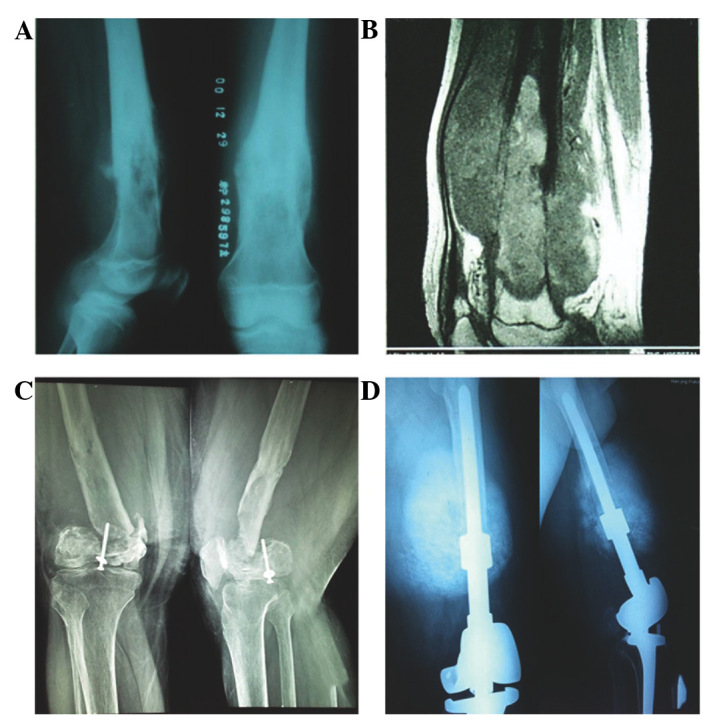
A male patient with late recurrent osteosarcoma 10 years post-surgery. (A) X-ray imaging showing osteoblastic destruction of the left distal femur, partial osteolytic changes, a visible periosteal reaction and a soft tissue mass. (B) MRI showing a mixed high and low signal intensity in the left distal femur, which formed a large soft tissue mass. (C) X-ray showing the allografting bone resorption and femoral condyle fragmentation at 8 years post-surgery. (D) X-ray imaging revealing a tumor shadow between the prosthesis and the host bone in the middle of the right femur with a soft tissue mass at 9 months after the second surgery. The lesion was confirmed to be an osteosarcoma by a post-operative pathological examination.

A 25-year-old male was admitted to the General Hospital of Nanjing Military Command on June 15, 2000, due to a gradually increasing, severe pain in the left knee that had lasted for one month. X-ray imaging (Fig. 2A) showed osteoblastic destruction of the left distal femur, partial osteolytic changes, a visible periosteal reaction and a soft tissue mass. MRI (Fig. 2B) showed a mixed high and low signal intensity in the left distal femur, which formed a large soft tissue mass. The patient was finally diagnosed with osteosarcoma of the left distal femur by biopsy and was administered two courses of high-dose methotrexate (HDMTX) and one course of ADR and DDP at the same dosage as previously reported (HDMTX, 10 g/m^2^; ADR, 60 mg/m^2^; and DDP, 120 mg/m^2^) (2). On August 16, 2000, the patient received an en bloc resection of the left distal femoral osteosarcoma and an allograft bone transplantation. The incision healed well. The same regimen as used in the pre-operative chemotherapy was applied post-operatively. One year after the surgery, the patient’s left knee was able to achieve 90 degrees of flexion. However, at six years post-surgery, the patient felt pain in the affected limb, which was now shortened and causing a limp. At eight years post-surgery, the patient had difficulty walking, felt exacerbated pain and the affected limb was shortened by 12 cm. A diagnosis of bone resorption following allograft bone transplantation was determined (Fig. 2C). In December 2009, the patient underwent a resection of the allograft bone and a reconstruction using a tumor prosthesis. Three months after the second surgery, the affected knee was able to achieve 45 degrees of flexion. In August 2010, X-ray imaging revealed a tumor shadow between the prosthesis and the host bone in the middle of the right femur, which was gradually increasing in size (Fig. 2D). In August 2011, the affected leg was amputated and the osteosarcoma was confirmed by a post-operative pathological examination. The patient was then provided with four courses of chemotherapy consisting of ADR, DDP and IFO (identical doses to previously). There were no recurrences or distant metastases within 10 months following the third surgery. The patient is currently being monitored using follow-up appointments.

### Case 3

**Figure 3.**
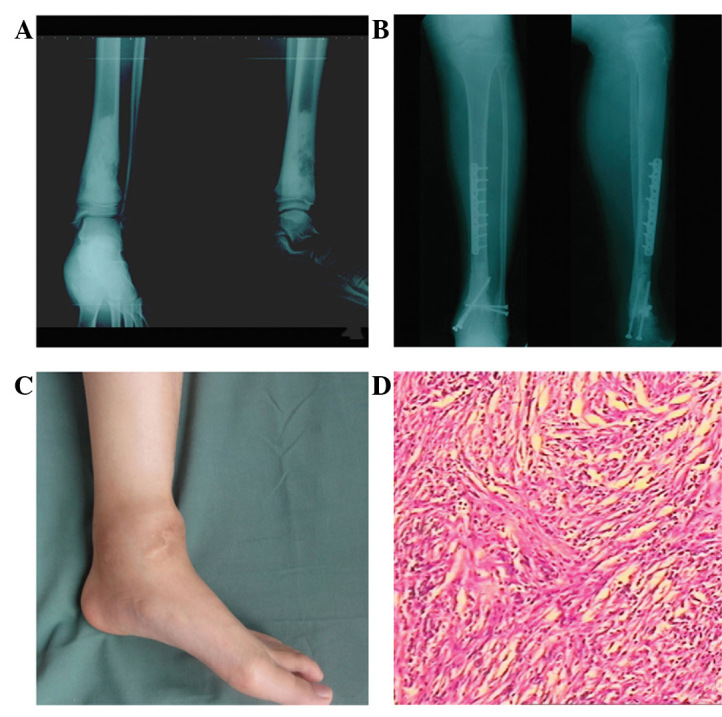
A female patient with late recurrent osteosarcoma 5 years post-surgery. (A) X-ray imaging showing osteoblastic destruction of the distal left tibia. (B) X-ray showing nonunion between the host bone and the distal side of the inactivated bone, accompanied by a posterior protrusion and varus deformity at 29 months post-surgery. (C) At 62 months subsequent to the initial surgery, the patient was hospitalized due to one month of ankle pain associated with a mass. (D) Post-operative pathological diagnosis demonstrating a fibroblastic osteosarcoma, identical to that diagnosed in the initial pre-operative examination (HE; magnification, ×10). HE, hematoxylin and eosin.

A 12-year-old female was admitted to Xinan Hospital of The Third Military Medical University on January 24, 2007 due to two months of intermittent lower left leg pain, which became aggravated in the second month and was accompanied by the growth of a mass. X-ray imaging showed osteoblastic bone destruction of the left distal tibia (Fig. 3A). MRI showed a mixed high and low signal intensity, with a visible periosteal reaction and an anterior soft tissue swelling. The patient was diagnosed with osteosarcoma of the distal left tibia by biopsy. The symptoms disappeared following one course of AP (ADR, 60 mg/m^2^; and DDP, 120 mg/m^2^) regimen chemotherapy. On March 2, 2007, the patient underwent a sectional removal of the osteosarcoma of the left distal tibia, a reconstruction using inactivated bone and an internal fixation, under general anesthesia. The post-operative pathological examination confirmed the initial diagnosis of osteosarcoma. Chemotherapy with the AP regimen (four courses with three week intervals) was initiated subsequent to the healing of the incision (two weeks post-surgery). An X-ray captured at 29 months post-surgery showed a nonunion between the host bone and the distal side of the inactivated bone, accompanied by a posterior protrusion and varus deformity (Fig. 3B). On July 31, 2009, the stainless steel plate-screw fixation was surgically removed from the left tibia. On April 4, 2012, 62 months after the initial surgery, the patient was hospitalized due to one month of ankle pain, which was associated with a mass (Fig. 3C). An X-ray examination demonstrated healing between the host bone and the proximal end of the inactivated bone, but a hypertrophic nonunion existed between the host bone and the distal end of the inactivated bone, which was accompanied by a posterior protrusion and varus deformity. Screw residues were left in the inferior tibia and fibula. MRI showed an anterior soft tissue mass in the lower left leg and osteolytic destruction of the left distal tibia, surrounded by edema. ECT showed an abnormal concentration of radionuclides in the left distal tibia, but no obvious abnormalities in the rest of the skeletal system. The patient was diagnosed with a recurrence of osteosarcoma of the left tibia. On May 4, 2012, an amputation of the middle section of the lower left leg was performed under epidural anesthesia. A post-operative pathological examination confirmed the pre-operative diagnosis of fibroblastic osteosarcoma (Fig. 3D). The incision stitches were removed at 14 days post-surgery, and the wound healed first time. There was no recurrence or metastasis at 6 months post-surgery. The patient is currently being monitored by follow-up appointments.

## Discussion

With the clinical application of neoadjuvant chemotherapy and the technical improvements in limb salvage surgery, the tumor-free survival rate of osteosarcoma has significantly improved. However, recurrence and metastasis occur in 1/3 of the affected patients and the treatment of these patients remains a challenge. According to a study by Spiegelberg *et al* (1), the rate of local recurrence following osteosarcoma surgery is generally 4–10%. However, the same rate in China is higher, at 10–20% (3). The recurrence of osteosarcoma generally occurs within less than two years post-surgery. Grimer *et al* (4) studied 96 patients with recurrent osteosarcoma and identified an average time of post-operative recurrence as 11 months (range, 1–66 months), during which, 60% of patients relapsed in less than one year post-surgery and 82% of patients relapsed in less than two years (5). Data from the Rizzoli Orthopaedic Institute (Bologna, Italy) indicated that the recurrence of osteosarcoma was significantly associated with the surgical margins and the effectiveness of pre-operative chemotherapy; if the surgical margin met the requirements, 97% of patients showed no local recurrence in 7 years, otherwise, the rate dropped to 71%. Following chemotherapy, the rate of local recurrence was 4% in patients with tumor cell necrosis >90%, otherwise, the rate increased to 10% (5).

Studies have indicated that a post-operative local recurrence of osteosarcoma may develop at 5 years, or even up to 20 years, post-surgery. The data from the Cooperative Osteosarcoma Study Group (COSS) (6) showed that between 1980 and 1998, only 23 (1.4%) of 1,702 cases suffered from a post-operative recurrence of osteosarcoma after five years (up to 14 years), which was defined as a late local recurrence of osteosarcoma. To the best of our knowledge, the present study has described 3 cases of recurrent osteosarcoma at 6.5, 10 and 5.2 years post-surgery, respectively, and is the first study of late local recurrence in Chinese patients.

The literature was searched using keywords including osteosarcoma, late local recurrence, limb and pelvic, and it was found that only 10 cases in the literature met the requirements for late local recurrent osteosarcoma (7–9). Therefore, to date, only a total of 13 patient cases, including those in the present study, have been reported (Table I).

These 13 patients consisted of eight males and five females. The average age of recurrence was 25.56 years (range, 13–42 years). A total of five cases involved the distal femur, the distal tibia and acetabulum were involved in two cases each and one case involved the proximal tibia (the locations of the recurrent lesions in the remaining cases were not described). The histological types of the recurrent tumors were as follows: three patients with fibroblastic-type, two with traditional-type, two with mixed-type, one with chondroblastic-type and one with telangiectasia-type osteosarcoma (the remaining cases were not described). The average time of post-operative recurrence was 10.02 years (range, 5.2–19.3 years). The treatment modalities were surgery with chemotherapy in five cases, surgery with radiotherapy in one case, surgery alone in two cases and one case did not complete the treatment (the remaining cases were not described). The average time of follow-up was 2.28 years (range, 0.5–4.7 years), excluding two cases that were not recorded. In total, six patients survived up to 4.5 years and 6 patients succumbed within the timespan of 0.6–4.7 years. The survival outcome was not recorded in one case.

The present data demonstrate that patients with late recurrent osteosarcomas are extremely rare, accounting for only 1% of the total osteosarcoma cases. The clinical manifestations do not exhibit evident specificity and are not significantly correlated with parameters that include the tumor location, histological type, efficacy of pre-operative chemotherapy and surgical approach (7). There is no consensus on the treatment plan for these patients, as this should be determined based on the general condition of the patients and the experience of the surgeon. Although a general post-operative chemotherapy regimen is applied, the drug toxicity during the treatment should also be monitored. In the present study, the cumulative dose of doxorubicin given to the patient in case 1 had reached the maximal tolerance, and the chemotherapy following the second surgery had to be terminated due to cardiac toxicity. The clinical outcomes of these patients were not ideal. In the present series of studies, out of the 12 patients who had a follow-up record, six cases survived and six succumbed to their condition. Evaluating the long-term prognoses of the these late recurrence osteosarcoma patients remains difficult due to the short follow-up times applied in the present study and literature cases. Whether neoadjuvant chemotherapy and surgical treatment may be applied to these late recurrence patients requires further study and observation.

The present series of data suggest that although the survival rate was continuously improved with the increased application of neoadjuvant chemotherapy and limb salvage surgery, attention should be focused on the long-term regular follow-up of these osteosarcoma patients. Certain researchers suggest that the follow-up time should be extended to 10 years (8), and that the patients should be followed up every 6 months from 5 years post-surgery. While it is of great importance to exclude the presence of lung metastases, more attention should be paid to the abnormal changes at the surgical site. Although routine ECT examinations are a viable way to exclude the presence of bone metastases, it is recommended that the patients in whom recurrence is suspected should be examined by PET/CT. One patient in the present study did not show an abnormality in the pre- or post-operative ECT, but the PET/CT examination confirmed the diagnosis of the post-operative pathology.

## Figures and Tables

**Table I.** Clinical characteristics of 13 patients with late recurrent osteosarcoma.

Patient No.	Ref.	Age at reccurrance (years)	Gender	Site	Histology	Pre-operative chemotherapy result	Recurrence time (years)	Retreatment	Follow-up (years)	Outcome
1	6	38.0	M	FD	Fib	Poor	7.5	NR	4.50	Survived
2	6	15.0	F	FD	Tel	Poor	5.5	NR	3.00	Succumbed
3	6	13.0	M	TD	CO	Good	5.3	NR	0.60	Survived
4	6	34.0	M	FD	Chb	Poor	8.5	NR	4.70	Succumbed
5	6	18.0	M	TP	Fib	Good	5.5	NR	3.90	Succumbed
6	7	24.7	M	NR	NR	NR	9.7	OP/CH	1.40	Succumbed
7	7	27.3	M	NR	NR	NR	11.3	OP/CH	3.60	Survived
8	7	32.3	M	NR	NR	NR	19.3	OP/CH	1.30	Succumbed
9	8	41.0	F	P	Chb/Ob	NR	17.0	OP/RA	NR	NR
10	1	42.0	F	P	Fib/Tel	NR	19.0	OP	NR	Succumbed
11	Study	13.0	F	FD	CO	Good	6.5	OP/CH	0.75	Survived
12	Study	35.0	M	FD	Ob	Good	10.0	OP/CH	0.80	Survived
13	Study	17.0	F	TD	Hb	NR	5.2	OP	0.50	Survived

Study refers to patients in the present study; FD, distal femur; TD, distal tibia; TP, proximal tibia; P, pelvis; Fib, fibroblast type; Tel, telangiectasia type; CO, traditional type; Chb, chondroblastic type; Ob, osteoblast cell type; OP/CH, surgery with chemotherapy; OP/RA, surgery with radiotherapy; OP, surgery alone; NR, not recorded; M, male; F, female.
